# Questioning the Universality of Mindfulness-Based Programs: Reflections From a Self-Construal Perspective

**DOI:** 10.3389/fpsyg.2022.908503

**Published:** 2022-06-14

**Authors:** Barry Tse

**Affiliations:** School of Social and Health Sciences, James Cook University, Singapore, Singapore

**Keywords:** mindfulness, self-compassion, self-construal, interdependent, independent, mindfulness-based programs, MBPs, cultures

## Introduction

Mindfulness is seeing unprecedented growth in popularity around the world. Apart from the ease of practice enabled by modern app-based mobile learnings and online courses, many organizations are making mindfulness part of organizational wellbeing programs for employees and stakeholders, with mindfulness classes now seen in different settings: schools, corporations, sports teams, and prisons. Amongst those on offer, many are considered popular Mindfulness-Based Programs (MBPs). These would include the Mindfulness-Based Stress Reduction (MBSR) by Kabat-Zinn (2005), the Mindfulness-Based Cognitive Therapy (MBCT) by Segal et al. (2012), originally for depression relapse prevention, and the Mindful Self-Compassion (MSC) by Neff and Germer (2018). Others are more context-specific, such as those dealing with mindful parenting (Coatsworth et al., 2015), mindful childbirth (Duncan and Bardacke, 2010; Bardacke, 2012), or mindful leadership (King and Badham, 2018; Lange and Rowold, 2019). There is always a program catering to different people's interests and situations. These programs are manualized, with fixed practices introduced weekly, and deviation from the program plan is strongly discouraged or prohibited to ensure the program remains intact no matter who or where it is delivered. When these programs are rolled out globally, they often assume the universality of their effectiveness and applicability across cultures and countries—an assumption based on the universality of dharma, as Williams and Kabat-Zinn (2011) put it.

Are the popular MBPs conceived and developed in a culturally neutral manner? From a historical perspective, though acknowledged as having their philosophical and practice roots in Buddhism, these programs are secularized, developed, or adapted into the current format and curricula for those living in Western societies (Karl et al., 2022). From Kabat-Zinn's (1982) original attempt to introduce mindfulness to chronic pain sufferers to the adaptation into a program for depression relapse prevention (Segal et al., 2012), the initial participants of these programs were those in the West. These “Western” formulated programs have gained popularity in the East as the mindfulness movement penetrates globally in the last decade or so, but no one knows for sure if these MBPs are equally effective when practiced in a different culture. Even within the United States, a recent systematic review of 94 randomized controlled trials (RCTs) revealed a lack of demographic diversity in samples skewed toward white, highly educated, middle-aged females (Eichel et al., 2021). This bias in demographics should be kept in mind before one can conclude the universality of the effect of mindfulness when applied to different cultures and demographic groups.

To complicate the issue further, definitions differ between secular mindfulness and its Buddhist origin. The Buddhist origin of *Samma Sati* (Right Mindfulness in the Pali language), one of the interrelated Noble Eightfold Paths from which secular mindfulness was adapted, encompasses remembering the Buddha, his teaching, and those who have achieved liberation before us (Bodhi, 2011, 2016). This definition is fundamentally different from the pure awareness of the present moment commonly used in secular mindfulness (Feldman and Kuyken, 2019).

In addition, as an evidence-based intervention, secular mindfulness takes a ritualistic bottom-up approach without the original top-down Buddhist ethics system as its moral compass (Karl et al., 2022). This missing moral code in secular mindfulness has already sparked intense debates and attracted criticism from those mindfulness scholars with a Buddhist background (Lindahl, 2015; Sharf, 2015; Bodhi, 2016; Purser, 2019). Buddhism being a widespread religion and faith tradition deeply woven into the Asian cultural fabric, could the understanding of mindfulness and its characteristics in Asia differ due to the long-embedded appreciation and understanding of Buddhist concepts?

The current measurements of mindfulness are largely conceptualized based or further expounded on the definition by Kabat-Zinn (2003), where, in a nutshell, mindfulness is the non-judgmental purposeful attention to the present moment. Examining how well these measurements work across cultures will help gauge whether the current understanding of mindfulness is universal. When looking at the two most commonly used mindfulness measurements, the Mindful Attention Awareness Scale (MAAS) by Brown and Ryan (2003) and the Five-Facet Mindfulness Questionnaire (FFMQ) by Baer et al. (2008), both did not demonstrate sufficient measurement invariance across cultural groups. Christopher et al. (2009) concluded from their study of the MAAS in Thailand and America that the scale only achieved partial scaler invariance, indicating a potentially different understanding of mindfulness between samples from the two countries. A more recent 16-country measurement invariance study by Karl et al. (2020) found that FFMQ did not achieve metric invariance. Their finding indicates the five-factor model fits better with samples from the Western individualistic cultures, while a six-factor model fits the samples from countries with a collectivistic culture. This study questions FFMQ's appropriateness when used in cross-cultural comparison, especially between the collectivistic East and the Individualistic West.

Will MBPs developed based on the same foundational concept of secular mindfulness able to maintain their efficacy when used in the collectivistic East? Perhaps, the preceding studies should have raised the alarm if the current popular MBPs could deliver their intended effect at the same level across different cultures.

## Inconsistent Effects of Mindfulness Across Cultures

Over the years, there has been a wealth of academic research supporting the benefits of mindfulness. Studies on the current secularized mindfulness practices have shown their ability to reduce stress and anxiety, and increase psychological wellbeing, despite varying degrees of effectiveness (Goldberg et al., 2021). Yet, mindfulness scholars may not have investigated if cultural factors may have played a role behind the variations. It was only until recently emerged a meta-analysis on mindfulness interventions' effectiveness amongst Chinese patients with psychosis (Tao et al., 2021). Although the study looked at 22 papers reporting on 19 studies from the mainland of China, Hong Kong, and Taiwan, it only provides cursory support for the benefits of mindfulness interventions on Chinese psychosis patients owing to the varying quality of the studies.

There were also inconsistencies when two or more cultures were compared. Ivtzan et al. (2018) tested an 8-week mindfulness program to promote flourishing *via* mindfulness in the United Kingdom and Hong Kong. Compared to the respective control groups, Hong Kong participants showed a decrease in the positive affect post-intervention, while UK participants demonstrated an increase. Another Singapore experiment also produced results counter to the outcome predicted by theory, and the study compares mindfulness, suppression, and reappraisal as ways to deal with sad moods. It discovered that those higher on Asian values found suppression achieved lower sad moods than mindfulness (Keng et al., 2017), a result not anticipated.

On the behavioral side, mindfulness has shown a small but significant effect on reducing intergroup bias (Oyler et al., 2021), and it could potentially increase cooperation in a negotiation situation (Masters-Waage et al., 2021). At the same time, mindfulness meditation is effective for some prosocial behaviors (Berry et al., 2020) and has a medium effect when used as an intervention (Donald et al., 2019). However, a meta-analytic study has put mindfulness or meditation's impact on prosocial behaviors at merely small to medium (Luberto et al., 2018). Kreplin et al. (2018) discovered that a significant increase in compassion after intervention only happened when the meditation trainer was one of the co-authors. Although the general direction of mindfulness contributing to prosocial behavior seems to have emerged, the effects are still inconsistent. However, the current systematic review appeared to only look at studies conducted primarily in the West. For example, only one study involved Chinese college students of the 26 studies examined by Luberto et al. (2018).

The lack of systematic reviews of mindfulness effectiveness taking a cultural lens prohibits a comprehensive understanding of how culture may affect intervention results. For example, a recently published study demonstrated that mindfulness had contrasting effects on prosocial behavior in people with different self-construal (Poulin et al., 2021). The study found that mindfulness meditation increased prosocial behavior by 40% for those with a stronger interdependent self-construal. However, for those who tended to perceive themselves as more independent (independent self-construal), mindfulness reduced prosocial behavior by 33%. This study even found opposite changes in prosocial behavior through its experimental design when subjects were primed for independent and interdependent self-construal. These findings of mindfulness making people behave more selfishly sound somewhat counterintuitive given the current view of mindfulness as a psychological intervention tool that promotes human flourishing. Nevertheless, the aim here is not to disprove the validity of mindfulness but to direct attention to the potential issue concerning how self-construal may potentially moderate and change the relationship between mindfulness and prosocial behaviors.

## Self-construal Could Be a Potential Issue

Markus and Kitayama's (1991) independent and interdependent self-construal is a frequently studied topic in cultural psychology. The concept simply looks at how the idea of “self” is construed. In a broad stroke generalization, people in Western countries most often see themselves as independent in self-construal, and people in East Asian countries often see themselves as interdependent and differ even in cognitive styles (Kitayama et al., 1997, 2019). It is essential to note that the practices and origin of mindfulness meditation come from the East, and the original mindfulness meditation in the Buddhist tradition would likely skew toward addressing those with an interdependent self-construal. When it found its new root in the West and was ushered into the field of psychological science, this Western adapted version of mindfulness meditation was designed in a way that has cleansed or tune-down of its interdependent cultural nuances.

In Buddhism, where mindfulness meditation originates, no separate and permanent self exists. Everything, including the concept of “I,” results from the working of causes, conditions, and finally ripening into effects or fruits. Each element is interdependent, and there is no way to locate an unchanging “I,” there is no fixed relationship with other elements, only “inter-relationship” and “co-existence”. Such Buddhist belief is referred to as “dependent origination” or “dependent co-arising” doctrine. It is mistaking relationships with things around us as something permanent or unchanging rather than constantly in a flux that leads us into various sufferings (de Silva, 2014). To put it simply, it is more of an antidote to the over-reliance on or over-identification of the interdependent self-construal variables that distorted their worldviews and impeded their social functioning. To demonstrate the impact of interdependence, a recent study in Hong Kong discovered a moderation effect that lower interdependent self-construal leads to more reliance on self-ability to cope, resulting in higher usage of reappraisal as a coping mechanism for dealing with an emotional situation (Chen and Cheung, 2021).

For Westerners who are more inclined to have an independent self-construal, what causes their psychological ailments are perhaps more to do with their attachment to the independent sense of self and over-identification with the idea of an independent self. Mindfulness's emphasis on looking past the interdependent relationship the concept of “I” has with the world and our mental processes may strengthen the idea of an independent self because the sense of an independent self becomes more prominent.

## Self-construal and Compassion

With the heightened sense of an independent self, self-blame, self-criticism, and self-judgment would become more apparent. For this reason, some practices in mindfulness aimed at self-care, such as loving-kindness and compassion, are prevalent and highly valued in MBPs. However, in the practice of compassion, the effect of self-construal would become more apparent. Criticism from Buddhist scholars has already questioned some popular programs' conceptualization, such as the focus on the “Self” in MSC practices (Anālayo and Dhammadinnā, 2021) not being congruent with the original Buddhist intention of reducing the sense of attachment to the concept of “self.” In the Buddhist tradition, the dedication of compassion is not directional or conditioned to the “self” but universal.

Just to illustrate and highlight the distinctive cultural differences when it comes to compassion, in a recent comparison of how Australians and Singaporeans express compassion, it was found that Singaporeans find it difficult to express care for others, and Australians find it difficult to express care for themselves (Steindl et al., 2019). Many MBPs will involve compassion practices that encompass devotion to self and others; if the ease of expressing compassion differs between countries or cultures, MBPs may have different effects when offered in different cultures. Perhaps, adjustments to the MBPs are needed to achieve the same goal. On the conceptualized mechanism of compassion, the current MSC program increases the sense of common humanity to help ease practitioners' sense of self-blame, self-criticism, over-identification, self-judgment, and isolation (Germer and Neff, 2019), assuming an opposite effect. However, an earlier study conducted in Thailand, Taiwan, and the US compared their self-construal, life satisfaction, and self-compassion (Neff et al., 2008) may demonstrate that common humanity is not exactly an antidote for self-judgment. In the study, apart from confirming that Thais are highest in self-compassion, followed by the Americans, and Taiwanese scored the lowest, the study also found that the Taiwanese have strong self-judgment yet high on common humanity amongst those with high interdependent self-construal.

Although evidence on compassion being affected by self-construal is far from sufficient, and focused studies comparing the cultural impact on the practice of compassion are limited, care should be taken to ensure the intended effects are not hampered.

## Conclusion

The current definition of mindfulness adopted by most MBPs is different from what was defined in its Buddhist origin. With mindfulness gaining popularity in Asia, where Buddhism is more widely understood and rooted in its cultural fabric, the different understanding of mindfulness makes potentially different operationalization of the concept. The different factor solutions and non-invariance of common mindfulness measurements applied to Asian samples have alarmed that attention needs to be paid to the potential cultural differences in the understanding of mindfulness.

At the program level, MBPs are designed to achieve specific aims. However, studies show potential differences in outcomes concerning programs run in Asia vs. the same program conducted in the West. However, empirical evidence making such exact comparisons is still scarce.

The cultural differences in self-construal between the interdependent East and independent West could play a role in the different effects of MBPs, as mindfulness' Buddhist doctrine seeks to help practitioners develop clarity in their understanding of the concept of “self”. However, the fundamental differences between the independent and interdependent self would mean that effective mechanisms could differ.

With the rapidly growing popularity and proliferation of various MBPs globally, mindfulness researchers may need to take a step back and revisit how mindfulness evolved into its current forms. It is perhaps also time for mindfulness researchers to question the assumed universality of MBPs, re-examine the cultural fit of mindfulness practices commonly deployed in different MBPs, and explore ways to ensure consistent effectiveness of MBPs when practiced in different cultures with different self-construal inclinations.

## Author Contributions

The author confirms being the sole contributor of this work and has approved it for publication.

## Conflict of Interest

The author declares that the research was conducted in the absence of any commercial or financial relationships that could be construed as a potential conflict of interest.

## Publisher's Note

All claims expressed in this article are solely those of the authors and do not necessarily represent those of their affiliated organizations, or those of the publisher, the editors and the reviewers. Any product that may be evaluated in this article, or claim that may be made by its manufacturer, is not guaranteed or endorsed by the publisher.
